# Dispersive white light continuum single Z-scan for rapid determination of degenerate two-photon absorption spectra

**DOI:** 10.1007/s00340-018-7011-0

**Published:** 2018-06-22

**Authors:** Aliasghar Ajami, Wolfgang Husinsky, Aleksandr Ovsianikov, Robert Liska

**Affiliations:** 1 Faculty of Physics, Semnan University, P. O. Box 35195-363, Semnan, Iran; 2 Institute of Applied Physics, TU Wien (Technische Universitat Wien), Wiedner Hauptstrasse. 8, 1060 Vienna, Austria; 3 Institute of Materials Science and Technology (E308), TU Wien (Technische Universitat Wien), Getreidemarkt 9, 1060 Vienna, Austria; 4 Institute of Applied Synthetic Chemistry, TU Wien (Technische Universitat Wien), Getreidemarkt 9, 1060 Vienna, Austria

## Abstract

We present an experimental technique to determine the degenerate two-photon absorption (2PA) spectra by performing a single Z-scan using a high-spectral-irradiance white light continuum (WLC) generated by a hollow core fiber. The hollow fiber was filled with Argon (Ar) gas at a pressure of 0.6 bar and was pumped with 500 mJ, 30 fs, and 800 nm pulses. The broadband WLC pulses with 350 nm bandwidth in the range of 600–950 nm were compressed to sub-8 fs pulses. To characterize and interpret the data obtained from this method, the spectral, temporal and spatial characteristics of the WLC were first analyzed. The WLC emerging from the compressor was dispersed using a prism pair and then focused into the sample by a cylindrical lens. Since different spectral components are spatially separated, any part of the sample in the beam cross section is irradiated with almost single wavelength pulses leading to only a degenerate 2PA process. The nonlinear transmittance was then measured by a charge-coupled-device (CCD) line camera as a function of the sample position while the sample was moved along the beam direction by a motorized translation stage. In this way the Z-scans at different wavelengths in the WLC spectral range can be measured and thus the wavelength-resolved degenerate 2PA spectra can be obtained by performing a single scan using dispersive WLC. This method was verified on a well-characterized dye Rhodamine B and yield a reasonable agreement with the data found in the literature. We used this method to determine the 2PA spectra of some two-photon initiators (2PIs) developed for two-photon polymerization (2PP) based 3D micro-structuring.

## Introduction

Two-photon absorption (2PA) is an optical nonlinear process in which two photons are simultaneously absorbed to excite the molecule to a higher energy level. Such a process is called degenerate when the frequencies of both absorbed photons are the same and nondegenerate if the two absorbed photons have different frequency. 2PA can be observed when the material is exposed to high enough intense light (e.g., ultrashort laser pulses) at wavelengths beyond the one-photon absorption (1PA) spectra. In the 2PA process the amount of absorbed light is proportional to the intensity square. Thus, the absorption of light drops drastically with radial and also axial distance from the focal point. It means that, only in a very small volume around the focal spot of a tightly focused pulsed laser beam the 2PA is substantially strong. By exploiting such a behavior it is feasible to modify the optical properties or induce a photolysis process in a very small volume within the bulk of a transparent material. For instance 3D micro-nano structuring with resolution beyond the diffraction limit of the 1PA can be achieved via two-photon polymerization (2PP) [1–3] and two-photon grafting (immobilizing molecules within the bulk of a polymer) [4, 5]. 2PA-based 3D data storage [6, 7] and 2PA-based photodynamic therapy [8, 9] are also two other well-known applications. Exploiting such promising applications has prompted researchers to undertake lots of efforts for developing new nonlinear materials such as organic chromophores with higher 2PA cross section [10, 11] and also establish new techniques for determination of the 2PA cross section with higher accuracy and precision.

2PA cross section of a material is an essential measure to assess the two-photon activity level of the nonlinear material in interaction with ultrashort laser pulses. Different methods have been proposed and utilized to determine the 2PA cross section among which the single wavelength Z-scan technique [12] has been used more widely due to its simplicity and accuracy. In this technique, the 2PA cross section is obtained for the specific wavelength delivered by the source used for the experiment.

The wavelength-resolved 2PA spectra are required for tuning the laser to the peak absorption in 2PA-based applications to enhance the efficiency. Additionally, for newly developed compounds the 2PA spectra are essentially necessary as a guide line for designing molecular with higher 2PA cross section [13]. To determine the 2PA spectra a tunable laser or a high intense white light continuum (WLC) source should replace the single wavelength source in conventional Z-scan. The following methods utilized for determining the 2PA spectra can be found in the literature.

- Using a tunable laser such as optical parametric amplifier (OPA) or optical parametric oscillator (OPO). The Z-scan can be repeated at different wavelengths by tuning the central wavelength of the laser output. From each Z-scan the 2PA cross section at the tuned wavelength can be determined. Applying this method yields to obtain the 2PA spectra via point-by-point fashion [14–18]. For instance, to obtain the 2PA spectra in the range of 600–950 nm with resolution of 10 nm the Z-scan experiment has to be repeated 35 times which is, of course, cumbersome and time-consuming.
- Using a WLC [19] source with a series of narrow band filters. In this method a narrow range of WLC spectrum is selected by placing a narrow band filter in front of the WLC beam. The transmitted beam through the filter, likely behaves as a single wavelength source, can be used to perform the Z-scan. By changing the filter and repeating the Z-scan at different wavelengths, the 2PA spectra can be obtained via point-by-point fashion which is also cumbersome and time-consuming [20–24].
- Using nondispersive WLC Z-scan. In this method the WLC beam is focused entirely in a single spot within the material using a spherical lens. In this way, the 2PA occurs via both degenerate and nondegenerate processes. As the sample is moved along the laser beam direction through the focal point, the nonlinear transmittance is measured by a spectrometer as a function of the sample position. Although, the measured Z-scans show the nonlinear transmittance for different wavelengths they comprise both degenerate and nondegenerate 2PA over the entire spectrum of the WLC with unknown contribution of each process. Therefore, it is not feasible to determine the pure degenerate 2PA spectra using this method [25–27]. However, this method delivers a simple way to obtain the wavelength-resolved nondegenerate 2PA cross section.
- Using dispersive WLC without scan. In this method the WLC beam is spatially dispersed and then focused by a spherical lens into the center of the sample. With this arrangement, different spectral components of the WLC beam are spatially separated from each other at the sample position and only degenerate TPA from the same spectral component can take place. By comparing the transmission of the sample at the focus to that of the pure solvent, the attenuation of different spectral components can be determined [28–30]. In this method the obtained 2PA spectrum is although of degenerate nature, it represents the relative, but not the absolute value of the 2PA cross section. It worth mentioning that, in this method, since the light beam is focused by a spherical lens, the spectral resolution within the sample (i.e., bandwidth existing per unit of length in the plane normal to the propagation direction) would change as a consequence of beam size variation if the sample is translated along the *z*-axis. Thus, the sample must be fixed at a position (preferably the focal point) and hence, this method cannot be considered as a Z-scan technique. Moreover, a separate Z-scan should be performed at a single wavelength since the 2PA cross section at a given wavelength should be known to convert the obtained relative spectrum to a spectrum showing the absolute 2PA cross sections.
- Using a dispersive WLC with a narrow slit. In this method a narrow slit is mounted on a stage so that the length of the slit is perpendicular to the dispersion direction of the WLC. By displacing the slit the wavelength of the transmitted light can simply be changed with an arbitrary amount. The transmitted narrow band laser beam can be utilized to carry out the Z-scan. In this way, by changing the position of the slit and repeating the Z-scan for the new wavelength the 2PA cross section can be measured over the entire WLC spectra via point-by-point determination scheme. Although this is a feasible method and similar to method (b) it has not been reported in literature.

The WLC can be generated by focusing high energetic short pulses into a transparent medium such as water [22], glass [31], optical fiber [32], photonic crystals [33], dielectric and semiconductors [34], crystals such as BaF_2_ [35] and inert gases such Krypton or Ar [36, 37]. We produced WLC by slightly focusing femtosecond pulses into a hollow fiber filled with Ar gas. In this paper a unique method, by which the absolute visible-to-near-infrared degenerate 2PA spectra can be determined via performing a dispersive WLC single Z-scan, is described in detail. This technique can be used for a rapid determination of the wavelength-resolved 2PA spectra of any nonlinear medium ranging from semiconductors to organic solutions. To check the validity of our technique over the WLC spectrum the 2PA cross section of a well-characterized organic dye, Rhodamine B [38, 39], was determined and the results are presented in this paper.

## WLC generation

**Fig. 1:**
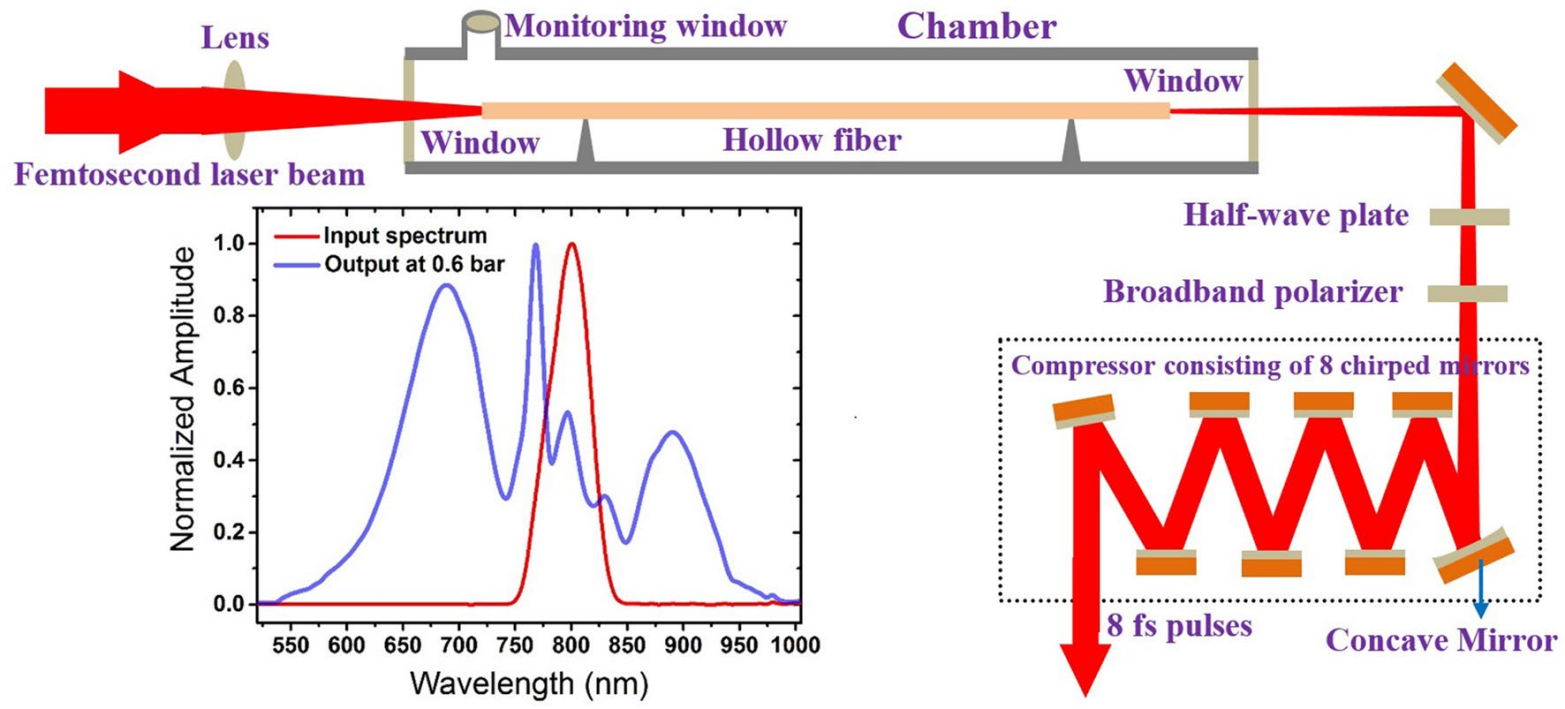
Setup used for WLC and sub-8 fs pulses generation

Figure 1 schematically shows the setup used for WLC generation and subsequently producing sub-8 fs pulses in this work. In this setup a compact pro Ti:sapphire laser system was used as a pump source for WLC generation. This system can produce 30 fs, 500 µJ pulses with repetition rate of 1 kHz at central wavelength of 800 nm. The pulses generated by this source are slightly focused using a 150 cm focal length plano-convex lens to a 190 µm diameter spot at the entrance of the 175 cm long hollow fiber mounted on a V-groove holder inside a chamber filled with Ar gas. The inner diameter of the hollow fiber (i.e., the capillary diameter) is 250 µm. Therefore, the proper mode matching between the input beam at the fiber entrance and the capillary radius (i.e., *w*_0_/*a* ≈ 2/3, where *w*_0_ is the laser spot radius at the fiber entrance and *a* is the capillary radius) [40] was achieved. In such a matching condition the single mode propagation through the hollow fiber can be obtained.

The chamber itself is mounted on a pair of x-y translation stages enabling the adjustment of the fiber axis so that the pump beam travels through the hollow fiber without striking the fiber wall. Since the beam spot stability is very crucial, the pump beam path from the amplifier output to the chamber entrance is covered to prevent air flow. The laser spot at the focus should possess a high-circularity to achieve the maximum efficiency of the WLC generation and also to attain a perfect beam profile demonstrating a spatially Gaussian distribution. To this end the focusing lens is mounted on a 2D rotation stage to adjust the incident angle on the focusing lens. The light guided to the exit of the hollow fiber is spectrally broadened as a consequence of self-phase modulation and very high-order nonlinear processes [36]. The efficiency of WLC generation in this setup is higher than 40% leading to output pulse energies of > 200 µJ which is very high for the purpose of 2PA measurements. To control the intensity of WLC required for measurements of different samples a set of half-wave plate and a polarizer is placed after the chamber. The transmitted WLC beam through the polarizer then travels through an ultra-broadband dispersive mirror compressor consisting of 8 chirped mirrors. The first mirror in the compressor is a concave mirror to re-collimate the incoming divergent beam.

**Fig. 2:**
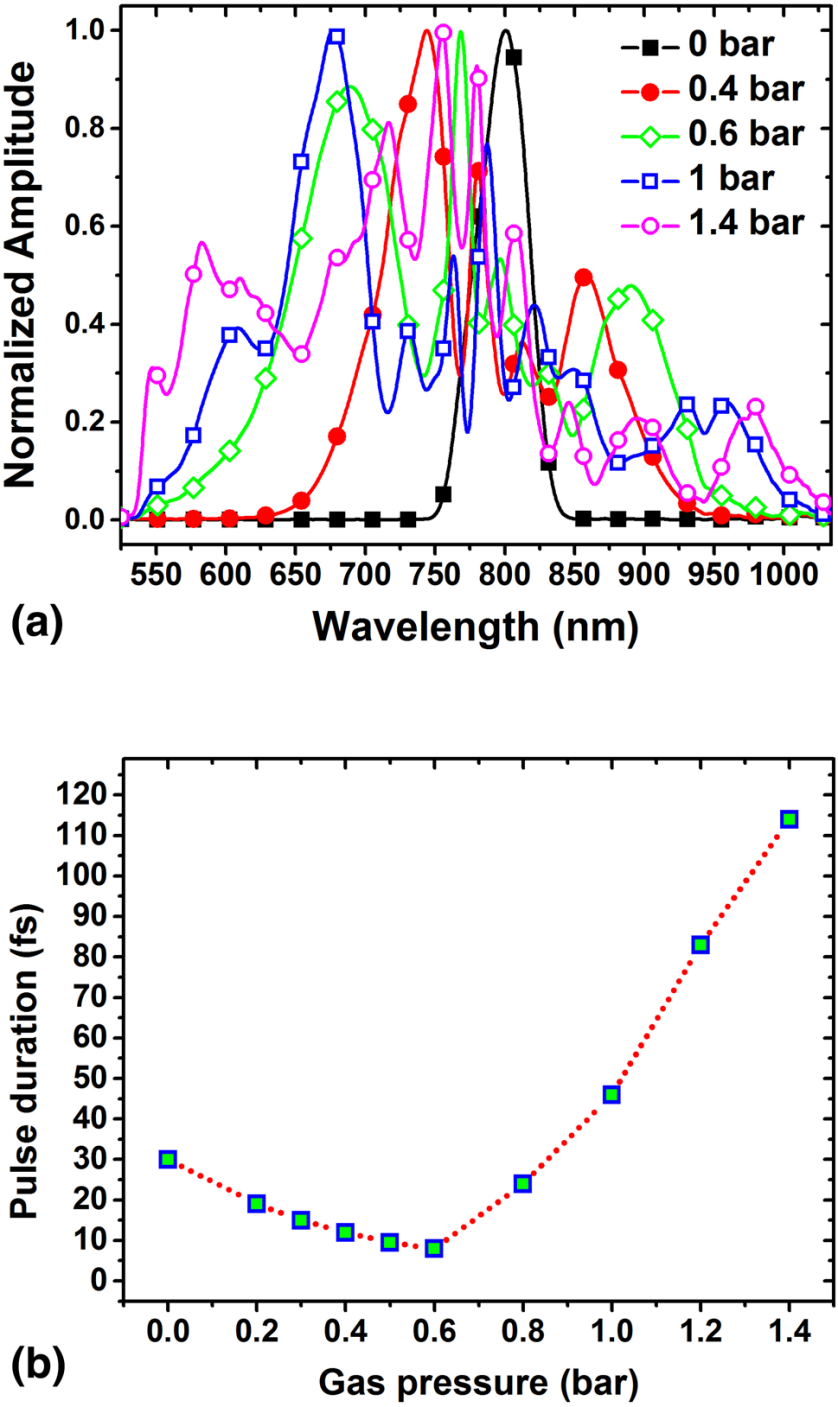
**a** Shows the WLC spectrum for different Ar gas pressure and **b** shows the pulse duration of the compressed pulses for different gas pressure

The spectrum bandwidth of WLC exiting the Hollow fiber depends strongly on the pressure of the Ar gas inside the chamber. Figure 2a shows how the bandwidth broadens as the gas pressure inside the chamber is increased. When the chamber is evacuated (i.e., zero pressure) the hollow fiber output is identical to the input pump (i.e., amplifier beam) with a peak at 800 nm and a bandwidth of 40 nm corresponding to a pulse duration of 30 fs. When the chamber is filled with Ar gas at a pressure of 0.4 bar two blue-shifted and red-shifted peaks appear at 745 and 860 nm, respectively. As the pressure of the Ar gas is increased to 0.6 bar the peaks shift to 690 and 890 nm. At the pressure of 1 bar the peak positions are 677 and 945 nm. When the pressure increased to 1.4 bar the peaks relocate to 585 and 980 nm so that the spectrum covers a large range of wavelengths from 500 to 1050 nm.

Pulse durations of the compressed pulses were measured after the compressor using an auto-correlator.

Figure 2b shows the measured pulse durations versus gas pressure inside the hollow fiber. The pulse duration decreased from 30 fs (for pump pulses) to about 8 fs as the pressure inside the Hollow fiber is increased from zero to 0.6 bar. By further increasing of the gas pressure inside the hollow fiber the spectral bandwidth of the WLC broadens, but the pulse width of the pulses emerging from the compressor does not accordingly decrease, as it is expected for Fourier-bandwidth-limited pulses. This happens because a total compensation of the chirp carried by the spectrally broadened pulses cannot be achieved by the compressor when the spectral bandwidth grows beyond the range of 600–950 nm. At a 0.6 bar gas pressure the chirped carried by the WLC can be completely removed by traveling through the compressor and thus leads to Fourier-transform-limited pulses possessing the shortest possible pulse width for the given bandwidth. At higher gas pressures, however, which results in broader WLC, the compressor is not capable to eliminate the whole chirp and thus the produced pulses are not bandwidth-limited. This was investigated by performing frequency-resolved optical gating (FROG). Fortunately the spectral range of 600–950 nm, corresponding to the shortest pulses of sub-8 fs, is the most desirable bandwidth in which very many of organic solutions such as two-photon initiators (2PIs) exhibit sensible 2PA.

## Dispersive WLC single Z-scan setup

**Fig. 3:**
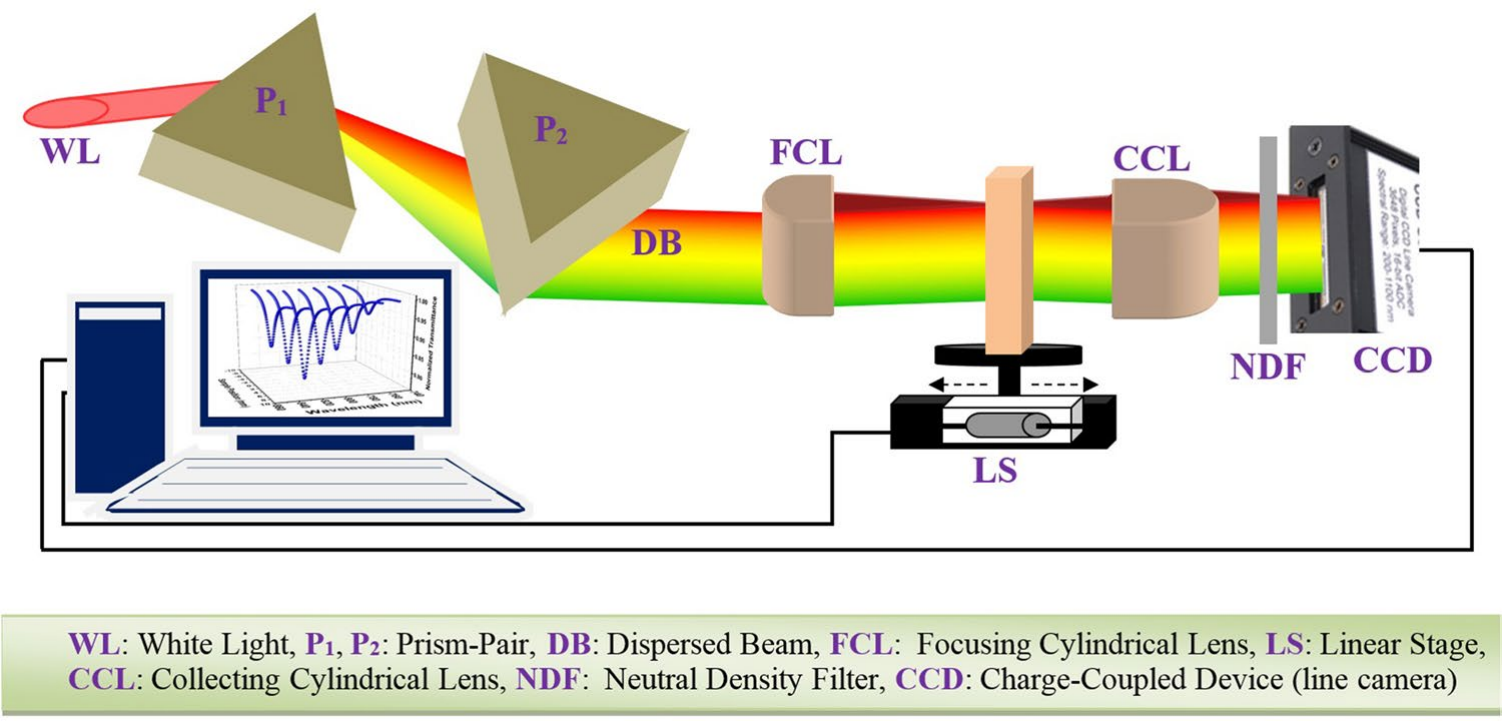
A tilted top-view of WLC Z-scan setup

The generated WLC consisting of sub-8 fs pulses was spatially dispersed in horizontal direction and then collimated using a F2-glass prism-pair separated by a distance of 140 cm in a parallel geometry (see Fig. 3). The incident angle for the input WLC beam was adjusted at Brewster angle (58.2°) to maintain the lowest loss due to the reflection from the prism surface. The collimated spatially dispersed beam (10 mm height and 30 mm width) was focused by a 150 mm focal length cylindrical lens into a 50 µm high (30 mm wide) line. In this way, the width of the beam remains unaffected. Thus, the spectral resolution (i.e., the wavelength interval within a certain width) does not vary allowing for scanning along the laser beam direction (i.e., performing Z-scan).

A cuvette (50 mm height, 50 mm width and 2 mm thick) containing the sample solution mounted on a step-motor translation stage is scanned along the propagation direction of the spatially dispersed focused beam through the focal plane (i.e., Z-scan). As the sample moves toward/away from the focal plan the height of the 30 mm wide beam reduces/increases leading to increasing/decreasing the intensity of each spectral component inside the sample and consequently results in higher/lower nonlinear absorption. Since in this method different spectral components of the WLC beam are spatially separated along a narrow horizontal line inside the sample, each infinitesimal volume within the illuminated region of the sample is irradiated with a narrowband (e.g., 10 nm) radiation leading to only degenerate 2PA from this same spectral component.

At each *Z*-position the transmitted intensity distribution is then imaged into a charge-coupled device (CCD) line camera [CCD-S3600-D from ALPHALAS (3600 pixels with pixel-width of 8 µm)] using a 50 mm focal length cylindrical lens. The transmittance as a function of sample position measured by each pixel of CCD line camera yields a Z-scan signal for the specific spectral component detected by that pixel. Thus, it is practically feasible to measure 3600 (number of pixels of the camera) Z-scan signals by performing only a single scan. To prevent saturation of the output signal of the CCD line camera an appropriate neutral density (ND) filter was used. It worth mentioning that, the transmission curves of ND filters are not perfectly uniform, but they vary as a function of wavelengths in the WLC spectrum. Furthermore, the transmission curves of various ND filters are not the same. Hence, the transmission of each ND filter [e.g., optical density (OD) of 0.3, 1 and 2] was first measured in the spectral range of 500–1100 nm using a Halogen lamp and a spectrometer (Ocean optic 400HC). The measured transmittance was then incorporated in calculating the spectral energy (i.e., energy existing in a given spectral region) as will be described in Sect. 5.2.

## Calibration of the CCD line camera

**Fig. 4:**
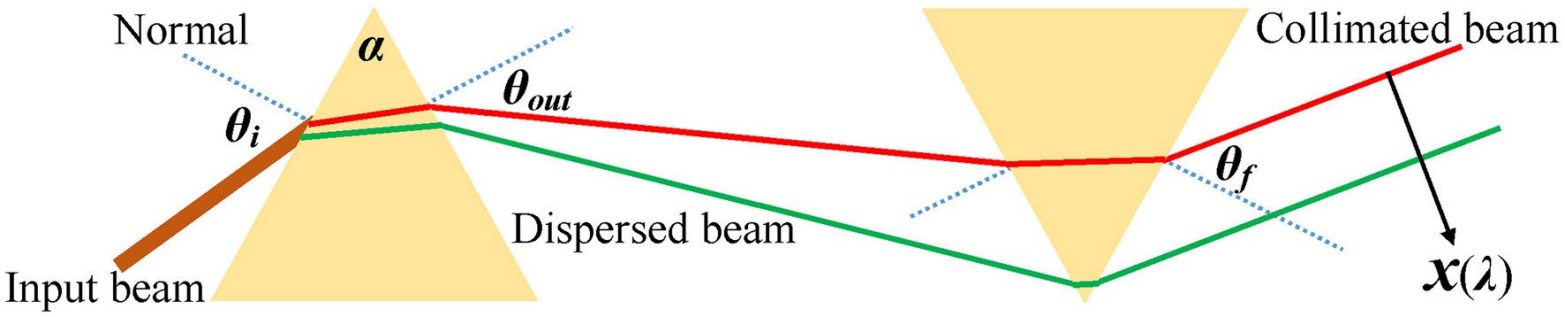
Dispersion and collimation of a broadband laser beam using a prism pair

The output signal of the CCD camera corresponds to a voltage (as a result of interaction of light with the detection area) versus pixel number. This must be converted to a signal representing the actual intensity versus wavelength. To convert the pixel number to wavelength (i.e., wavelength calibration) both calculations and experimental measurements were carried out. Using a prism pair in parallel configuration, as shown in Fig. 4, the output angle from the first and second prism (*θ*_out_ and *θ*_f_ respectively) and the spectral position *x*(*λ*) in the direction perpendicular to the collimated dispersed beam are given by Eqs. (1–3).

1 $${\theta _{{\text{out}}}}=\arcsin \left[ {n\,(\lambda )\,\sin \left[ {\alpha - \arcsin \left[ {\frac{{\sin \,({\theta _i})}}{{n\,(\lambda )}}} \right]} \right]} \right]$$

2 $${\theta _{\text{f}}}=\arcsin \left[ {n\,(\lambda )\,\sin \left[ {\alpha - \arcsin \left[ {\frac{{\sin \,({\theta _{{\text{out}}}})}}{{n\,(\lambda )}}} \right]} \right]} \right]$$

3 $$x\,(\lambda )=\left( {d\;{\theta _{{\text{out}}}}} \right)\left( {\frac{{{\theta _{{\text{out}}}}}}{{{\theta _{\text{f}}}}}} \right),$$

where *θ*_*i*_ is the incident angle, *α* is the apex angle of the prism, *d* is the distance between two prisms in parallel scheme and *n*(*λ*) is the wavelength dependent refractive index of the f2-glass given by Eq. 4 [41].

4 $$n\,(\lambda )={\left( {1+\frac{{1.34533359{\lambda ^2}}}{{{\lambda ^2} - 0.00997743871}}+\frac{{0.209073176{\lambda ^2}}}{{{\lambda ^2} - 0.0470450767}}+\frac{{0.937357162{\lambda ^2}}}{{{\lambda ^2} - 111.886764}}} \right)^{{\raise0.7ex\hbox{$1$} \!\mathord{\left/ {\vphantom {1 2}}\right.\kern-0pt}\!\lower0.7ex\hbox{$2$}}}}$$

**Fig. 5:**
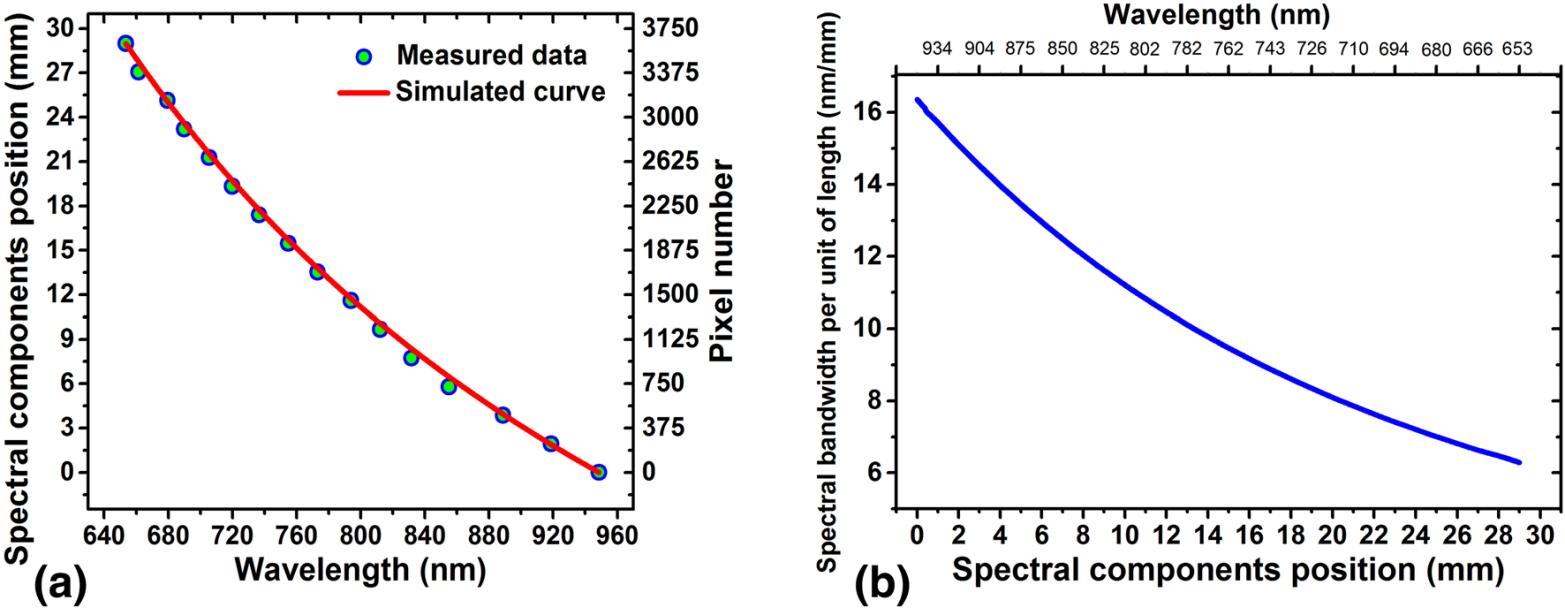
**a** Shows the spatial position of different component of a dispersed WLC along 30 mm line; circle points represent measured data and the solid line indicates the calculation results when F2-glass prism pair with separation distance of 140 cm was employed. **b** Illustrates the spectral bandwidth exists in 1 mm length along the dispersion direction versus position (or corresponding central wavelength)

In Fig. 5a the solid line exhibits the results calculated by Eq. (3). The calculation reveals how the spectral components are distributed over the 29 mm long detection area of the CCD line camera (i.e., along the dispersion direction; perpendicular to the beam propagation direction) after the second prism. The distance between two prisms was assumed 140 cm as identical to the actual distance in the used setup. The points in Fig. 5a indicate the experimental data measured by a spectrometer. To do this, the spectrometer mounted on a translation stage was displaced along the dispersion direction, perpendicular to the beam propagation direction, in a step size of 2 mm. As can be seen from Fig. 5a the absolute slop of the plot reduces versus wavelength indicating the fact that at shorter wavelength each unit of spectrum bandwidth is distributed over a longer spatial length along the dispersion direction. This leads to higher spectral resolution for the CCD line camera in the shorter wavelength region. This conclusion is shown more illustratively in Fig. 5b where the spectrum bandwidth in units of length of the CCD line camera is plotted versus the spectral position measured with respect to that end of the CCD camera where exposed to the longest wavelength in the used WLC spectrum (950 nm in this case). From Fig. 5b it is clearly seen that the spectral bandwidth in unit of length is larger for the region where exposed to longer wavelength. In other words, at longer wavelength the CCD line camera displays lower spectral resolution. The results of the computation, which were confirmed experimentally, indicate that the spectral resolution at a wavelength of 650 nm is almost three times higher than the resolution at a wavelength of 950 nm.

**Fig. 6:**
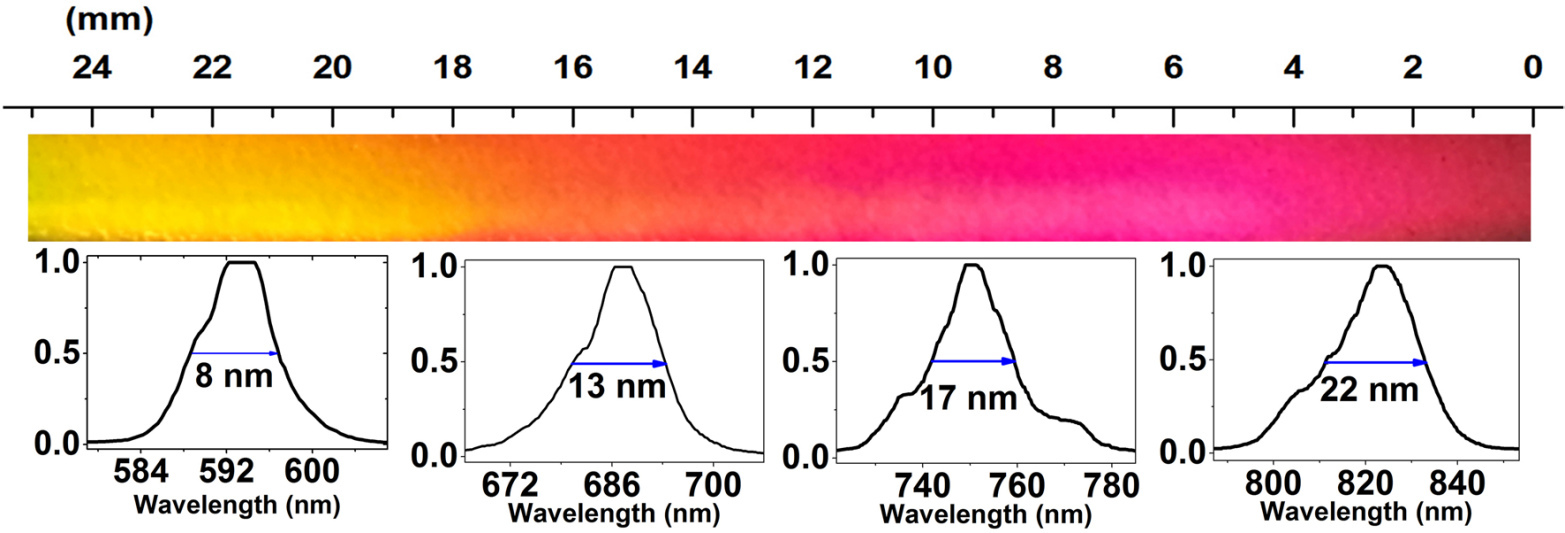
Spectral bandwidth measured at different spectral positions (wavelengths)

In Fig. 6 a photo of a part of the dispersed WLC along with a few measured spectrums at different spectral positions are shown. The bandwidth measured directly with the spectrometer clearly demonstrates a higher resolution at shorter wavelengths.

By interpolating the data presented in Fig. 5a each pixel number can simply be converted to the corresponding wavelength and thus the wavelength calibration of the CCD line camera can be achieved.

In this method as the sample is scanned 20 mm in step size of 0.25 mm along the beam, the spectrum of the transmitted spatially dispersed WLC is measured at each z-position. Therefore, the ultimate measured data represent a matrix of *NM* dimension. In this notation *N* denotes the total number of pixels of the CCD line camera (i.e., 3600) and *M* denotes the number of *z*-positions (i.e., 81) during the entire Z-scan experiment. Therefore, each element in this large dataset is defined with two indices of wavelength and *z*-position.

From Fig. 5a, which shows the pixel number versus wavelength, it can be ascertained that the spectral bandwidth existed on an individual pixel varies from 0.04 nm at 650 nm wavelength to about 0.13 nm at 950 nm wavelength giving an average spectral resolution of almost 0.08 nm. It is understood that such a small step size is not practically required in determining the 2PA cross section. Therefore, in the real experiment the resolution was reduced from 0.08 to 5 nm. This was done by adding the signals receiving from 60 pixels that leads to transform the 3600 × 81 matrix to a more compact matrix of just 60 × 81. This also results in increasing the signal to noise ratio.

## 2PA spectra determination

By rearranging the new compact matrix of data (i.e., converting the 60 × 81 matrix into 81 × 60 matrix) the nonlinear transmission of each particular spectral region can be obtained for all -positions giving rise to attain 60 Z-scan signals for different examined regions ranging from 650 to 950 nm. The 2PA cross section at each wavelength then can be extracted by fitting Eq. 5 to that respective Z-scan.

5 $$T(z)=\sum\limits_{{n\,=\,0}}^{\infty } {\,\frac{{{{\left( { - \left( {{{{\sigma _2}\,\lambda \,{N_{\text{A}}}\,\rho \times {{10}^{ - 3}}} \mathord{\left/ {\vphantom {{{\sigma _2}\,\lambda \,{N_{\text{A}}}\,\rho \times {{10}^{ - 3}}} {h\,c\,\,}}} \right. \kern-0pt} {h\,c\,\,}}} \right)\,L\,{I_0}} \right)}^{n}}}}{{\,{{\left( {n+1} \right)}^{\,{\raise0.7ex\hbox{$3$} \!\mathord{\left/ {\vphantom {3 2}}\right.\kern-0pt}\!\lower0.7ex\hbox{$2$}}}}\,{{\left( {1+\frac{{{z^{\,2}}}}{{z_{{\text{R}}}^{{\,2}}}}\,} \right)}^{n}}\,}}} ,$$

where *T* is the normalized transmittance, *L* is the sample thickness, *Z*_R_ is the Rayleigh range, *z* is the sample position measured with respect to the focal plane, *I*_0_ is the peak on-axis intensity at the focal plane, *h* is the Plank constant, *c* is the light speed in free space, *N*_A_ is the Avogadro constant, *ρ* is the concentration of the examined solution in mole/lit, *λ* is the wavelength and *σ*_2_ is the 2PA cross section.

To extract the 2PA cross section from fitting Eq. 5 to each Z-scan the magnitude of the respective *I*_0_ must be known. To this end, variables such as pulse duration, beam waist and the spectral energy must be known.

### Beam and pulse characterization of the dispersed WLC

**Fig. 7:**
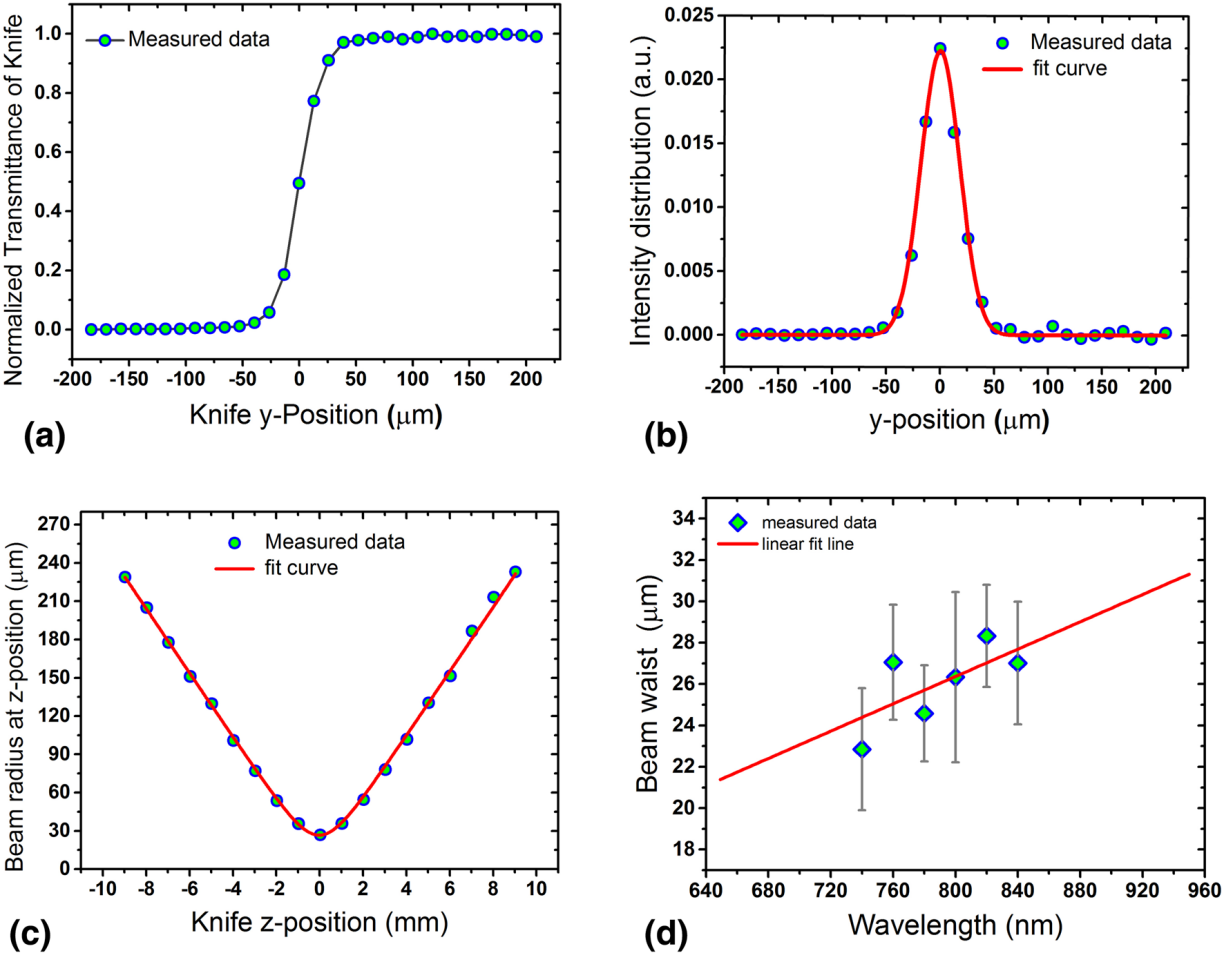
Different steps for determining the beam waist radius of different beams. **a** Shows the knife-edge transmittance, **b** shows the radial intensity distribution, **c** shows the beam radius at different *z*-position measured with respect to the waist position and **d** shows the beam waist radius for different beams having various wavelengths

The wavelength dependent beam waist radius was determined experimentally. To this end, a narrow band spectral range was selected at a few different wavelengths by placing a narrow slit in front of the collimated dispersed WLC beam. The waist of the selected part of the dispersed beam was measured using the knife-edge technique. The knife was mounted on a two-dimensional (*y*-*z*) motorized translation stage. *z*-position is measured with respect to the focal plane and *y*-position is measured with respect to the beam axis (center). At each *z*-position the knife was scanned along the *y*-direction (normal to the beam propagation direction) while the transmitted energy was measured as a function of the knife position in *y*-direction. As an example, Fig. 7a shows the knife transmittance versus *y*-position at *z*-position = 1 mm (i.e., 1 mm away from the waist position) for 800 nm wavelength. Figure 5b shows the derivative of data shown in Fig. 7a that yields the spatial intensity distribution in *y*-direction. The solid line in Fig. 7b indicate the Gaussian fit $I(y)={I_0}\,\exp ({{ - 2{y^2}} \mathord{\left/ {\vphantom {{ - 2{y^2}} {w{{(z)}^2})}}} \right. \kern-0pt} {w{{(z)}^2})}}$ to the data from which the beam radius *w*(*z*) at the given *z*-position = 1 mm was extracted 36 µm. By repeating this procedure at different *z*-positions for pre-focus and post-focus locations the beam radius at different *z*-positions was obtained. As an example, Fig. 7c shows the beam radius versus *z*-position for 800 nm wavelength. The solid line in Fig. 7c represents the fit curve to the measured data using Eq. 6 from which the beam waist radius was determined 27 µm.

6 $$w{(z)^2}={w_0}^{2}\left( {1+\frac{{{z^2}}}{{{z_0}^{2}}}} \right),$$

where *w*(*z*) is the beam radius at position *z, w*_0_ is the beam waist radius, *z*_0_ is the Rayleigh length and *z* in the position measured with respect to the waist position.

In this way the beam waist radius was measured for several wavelengths as shown in Fig. 7d. The entire measurements including the knife scanning in *y*-direction, moving the knife in *z*-direction, detecting the transmittance, calculating the derivative of the transmittance data and fitting processes for both beam radius and beam waist extraction were handled using a Labview program. From the theory of the focused Gaussian beam it is known that the beam waist changes linearly with the wavelength according to Eq. 7. Therefore, by extrapolating the fit line in Fig. 7d the beam waist at other wavelengths can be estimated.

7 $${w_0}=\frac{{2\,M{}^{2}\,\lambda \,f}}{{\pi \,D}},$$

where *M*^2^ is the beam quality factor, *λ* is the wavelength, *f* is the focal length of the focusing lens and *D* is the beam size on the focusing lens.

The pulse duration at different wavelengths was measured by an auto-correlator. Within an experimental error of 12% the pulse duration can be considered equal to 100 fs for all different wavelengths in the range of 650–950 nm.

### Determining the spectral energy

The spectral energy can be directly extracted from the spectrum measured with the CCD line camera if the outcome of each camera pixel is proportional to the light intensity received by the camera. Since the camera sensitivity and the ND filter transmission are both wavelength dependent, the spectrum measured with the CCD line camera was calibrated incorporating the camera sensitivity curve (delivered by the company) and the ND filter transmission curved measured experimentally in this work.

Performing the wavelength and intensity calibration, the CCD signal consequently yields the actual WLC spectrum (i.e., the intensity in an arbitrary unit versus the wavelength). Then, the spectral energy can be determined using (dI/Δ*I*)*E* where dI is the summation of intensity detected by each adjacent 60 pixels and Δ*I* is the summation of intensity detected by all 3600 pixels when the sample is far away from the focal plane where the nonlinear absorption is negligible. *E* is the net pulse energy received by the sample. By measuring the entire pulse energy prior to the prism pair and subtracting the loss due to the reflection from the surface of prisms as well as the focusing lens the value of *E* is obtained. With the spectral energy, the beam waist radius at each wavelength and the pulse duration the magnitude of *I*_0_ relevant to each Z-scan can now be calculated. By substituting the value of *I*_0_ in Eq. 1 the 2PA cross section at each wavelength can be extracted by fitting this equation to the relevant Z-scan data.

The entire measurement including the sample scanning, detecting the transmittance at each sample position by a CCD line camera, dividing the WLC spectrum into any number of region with arbitrary bandwidth, rearranging the data to determine the Z-scan for each specific spectra region, fitting the theoretical curve to the experimental data, extracting the 2PA cross section at each wavelength and then plotting the 2PA spectra is performed by a LabVIEW program. Determining the 2PA spectra is now as easy as the linear absorption spectra using the proposed method including our designed program. When the setup is well aligned, after mounting the sample on the translation stage, setting the concentration of solution and WLC pulse energy in the program and then running the program the 2PA spectra with an arbitrary resolution will be displayed on the monitor in less than 2 min.

**Fig. 8:**
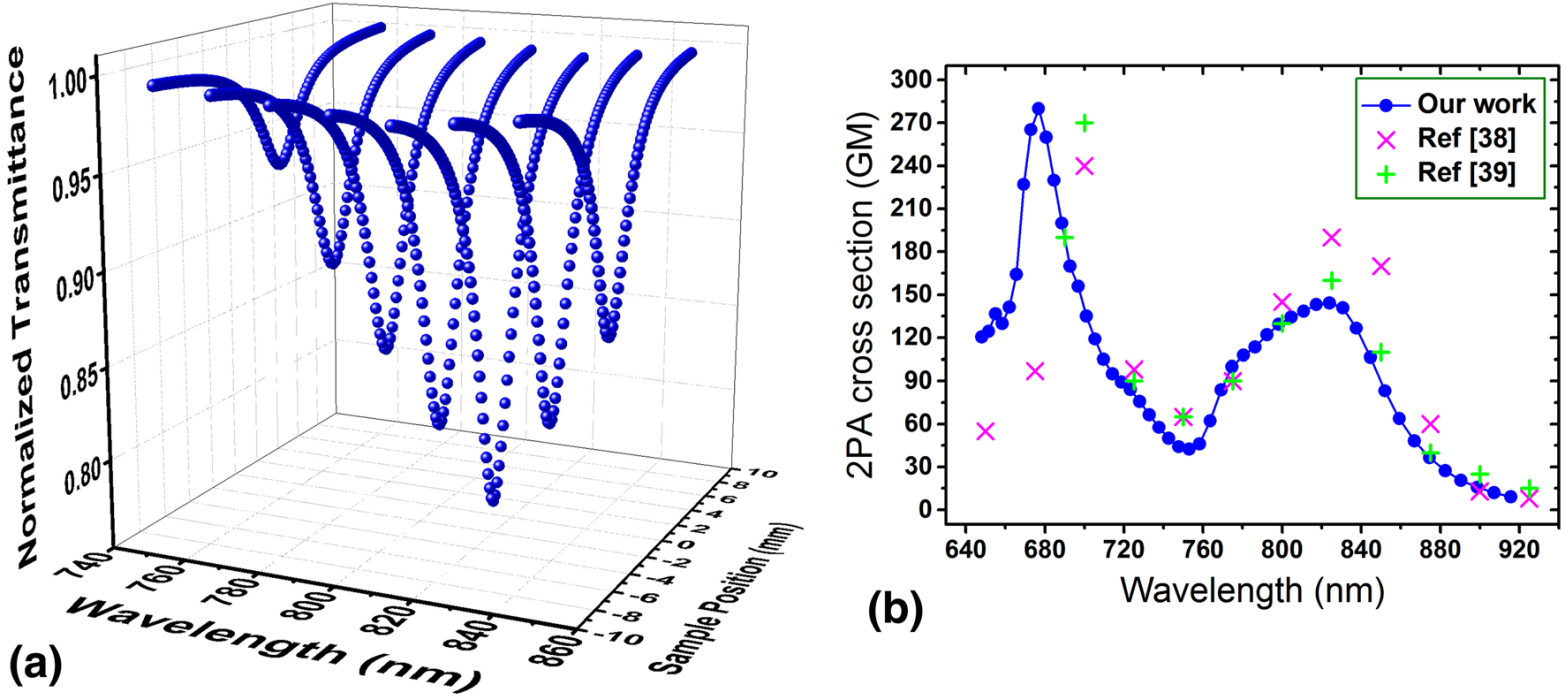
**A** Represents a few selected Z-scans (fit data) at different wavelengths. **B** Indicates the 2PA spectra of Rhodamine B in the spectral range of 650–930 nm

To test and verify this method Rhodamine B was examined as one of the few materials for which the 2PA spectra can be found in the literature. In Fig. 8a a few Z-scans (e.g., fit data) at some selected wavelength are shown. Figure 8b shows the 2PA spectra of Rhodamine B indicating a peak 2PA around 820 nm. The solid circles shows the data measured in our work and the plus and cross symbols demonstrate the data from the literature [38, 39]. The data obtained in our work shows a reasonable agreement with the data presented in the literature proving that our proposed method is a reliable technique for the rapid determination of the 2PA spectra. We used this method to determine the 2PA spectra of a few newly developed 2PIs which has been used for 2PP at different wavelengths [42].

## Conclusion

We generated a high-spectral-irradiance WLC by slightly focusing femtosecond pulses into a hollow core fiber filled with Ar gas. This WLC can be used as an ultra-broadband source in *Z*-scan experiments for rapid and simultaneous measurements of the degenerate 2PA spectrum of verity of nonlinear mediums. Depending on the Ar gas pressure the spectral bandwidth of the WLC broadens by more than 500 nm in the range of 500–1000 nm. The produced WLC pulses were compressed to sub-8 fs pulses using a compressor consisting of 8 chirped mirrors. The WLC beam emerging from the compressor was dispersed and collimated using a prism pair. The collimated dispersed beam was focused by a cylindrical lens thus, the optical pathways for different spectral components of the focused beam were spatially separated from each other inside the sample leading to the 2PA process of only degenerate nature. The beam through the sample is imaged into a CCD line camera which yields the Z-scans for different spectral component in the range 650–950 nm. In this way the degenerate 2PA spectra of the examined material can be determined via a single scan using a dispersed WLC. We experimentally verified this method on a well-characterized dye Rhodamine B. The reasonable agreement between our results and the results found in the literature proves the reliability and usefulness of this technique. This technique establishes the fastest method for determining the wavelength-resolved absolute values of the 2PA cross section (i.e., 2PA spectra) by performing a single Z-scan using a dispersed WLC. We used this method to measure the 2PA spectra of a few newly developed 2PIs which have been used for 2PP at different wavelengths.
